# OR9-001 - Exome sequencing in monogenic Behçet-like disease

**DOI:** 10.1186/1546-0096-11-S1-A184

**Published:** 2013-11-08

**Authors:** Q Zhou, R Laxer, M Pelletier, M Ramaswamy, H-Y Wang, D Chin, A Gül, C Sibley, M Barat-Houari, R Siegel, DL Kastner, I Aksentijevich

**Affiliations:** 1 Inflammatory Disease Section, National Human Genome Research Institute (NHGRI/NIH), Bethesda, USA; 2 Hospital for Sick Children, Toronto, Canada; 3 Immunoregulation Section, National Institute of Arthritis and Musculoskeletal and Skin (NIAMS/NIH), USA; 4 Laboratory of Host Defenses, National Institute of Allergy and Infectious Diseases (NIAID/NIH), Bethesda, USA; 5 Istanbul Faculty of Medicine, Istanbul University, Istanbul, Turkey; 6 Office of Clinical Director, National Institute of Arthritis and Musculoskeletal and Skin (NIAMS/NIH), Bethesda, USA; 7 Département de Génétique, LBM -CHU de Montpellier, Montpellier, France

## Introduction

A Caucasian family with an affected mother and 2 affected daughters presented with early onset Behçet–like disease, manifesting with arthralgia/arthritis, mouth and genital ulcers, and uveitis. They are negative for HLA*B51. Their symptoms are significantly ameliorated with TNF-inhibitors.

## Objectives

To identify the causative mutation(s) in this family we sequenced the exomes of the 3 affected patients.

## Methods

Exome data were filtered for novel variants not present in dbSNP, the 1000 Genomes Pilot Project, NHLBI Exome Sequencing Project (ESP5400), and 124 exomes from in-house data.

## Results

We identified 21 putative candidate variants that are both novel and consistent with dominant inheritance. Sanger sequencing validated all 21 variants. Three variants identified in *TNFRSF9*, *MGEA5*, and *TNFAIP3* genes were confirmed to have arisen *de novo* in the affected mother based on the genotyping of the healthy maternal grandmother, the maternal unaffected brother, and the unaffected paternal aunt. The candidate variant in *MGEA5* was predicted as benign by PolyPhen-2. The 2 candidate variants that remained for consideration are p.C78W (*TNFRSF9;* CD137; 4-1BB) and p.L227X (*TNFAIP3;*A20). Haplotype analysis showed that p.C78W occurred *de novo* on the haplotype inherited from the grandmother. The p.L227X mutation-associated haplotype was found on the grandfather’s haplotype; his sample is not available for analysis. Because we reached the limit for further analysis of candidate variants in the family, we studied 6 Turkish familial Behçet cases and 56 sporadic Caucasian cases. All of these individuals were negative for mutations in either candidate gene. Both 4-1BB and A20 are strong candidates and potentially in the same signaling pathway. 4-1BB is a TNF-family receptor that costimulates T cell responses and promotes survival of lymphocytes and dendritic cells; A20 is a negative regulator of NF-kB activation by TNF and TLR family receptors. Mutant 4-1BB expressed in Jurkat cells was associated with reduced expression on the plasma membrane, and activated T cells from all 3 patients had a marked decrease in surface expression, especially in CD8^+^ T cells. Despite reduced surface expression, the C78W TNFRSF9 mutation could have a gain-of-function phenotype like TNFR1 mutations in TRAPS, leading to intracellular retention of mutant protein and spontaneous signaling, which in this case could amplify immune responses by T cells and other cell types expressing 4-1BB. Patient peripheral blood cells also had lower total A20 protein levels relative to controls and, consistent with A20 lack of function, increased I-κB degradation after cellular activation.

## Conclusion

Behçet-like disease in this family is associated with mutations in 2 genes that affect immune cell survival and production of inflammatory cytokines. Additional functional studies will determine whether 1 or both mutations may contribute to this dominantly-inherited phenotype.

## Competing interests

None declared.

